# PTMD: A Database of Human Disease-associated Post-translational Modifications

**DOI:** 10.1016/j.gpb.2018.06.004

**Published:** 2018-09-21

**Authors:** Haodong Xu, Yongbo Wang, Shaofeng Lin, Wankun Deng, Di Peng, Qinghua Cui, Yu Xue

**Affiliations:** 1 Department of Bioinformatics & Systems Biology, MOE Key Laboratory of Molecular Biophysics, College of Life Science and Technology and the Collaborative Innovation Center for Biomedical Engineering, Huazhong University of Science and Technology, Wuhan 430074, China; 2 Department of Biomedical Informatics, School of Basic Medical Sciences, MOE Key Laboratory of Molecular Cardiovascular Sciences, Center for Non-coding RNA Medicine, Peking University, Beijing 100191, China

**Keywords:** Posttranslational modification, Phosphorylation, PTM–disease association, Disease–gene network, AKT1

## Abstract

Various **posttranslational modifications** (PTMs) participate in nearly all aspects of biological processes by regulating protein functions, and aberrant states of PTMs are frequently implicated in human diseases. Therefore, an integral resource of **PTM–disease associations** (PDAs) would be a great help for both academic research and clinical use. In this work, we reported PTMD, a well-curated database containing PTMs that are associated with human diseases. We manually collected 1950 known PDAs in 749 proteins for 23 types of PTMs and 275 types of diseases from the literature. Database analyses show that **phosphorylation** has the largest number of disease associations, whereas neurologic diseases have the largest number of PTM associations. We classified all known PDAs into six classes according to the PTM status in diseases and demonstrated that the upregulation and presence of PTM events account for a predominant proportion of disease-associated PTM events. By reconstructing a **disease–gene network**, we observed that breast cancers have the largest number of associated PTMs and AKT1 has the largest number of PTMs connected to diseases. Finally, the PTMD database was developed with detailed annotations and can be a useful resource for further analyzing the relations between PTMs and human diseases. PTMD is freely accessible at http://ptmd.biocuckoo.org.

## Introduction

Posttranslational modifications (PTMs) are essential biochemical reactions that covalently regulate the conformation, activity, and stability of proteins, and play a critical role in a broad spectrum of biological processes [1], [2]. For example, phosphorylation is strongly implicated in orchestrating signal transduction, cytoskeleton rearrangement, and cell cycle progression [3], [4]. Additionally, acetylation regulates the transcription and cellular metabolism through the modification of histones and nonhistone proteins [5], [6], whereas ubiquitination usually mediates the proteasomal degradation process by conjugating poly-ubiquitin chains to target proteins [7], [8]. Currently, over 620 types of PTMs have been experimentally discovered (http://www.uniprot.org/docs/ptmlist.txt), while accumulating evidence has revealed that the abnormal status of PTMs is frequently involved in various human diseases, such as cancers, diabetes, and neurodegenerative diseases [9], [10], [11]. For example, the ubiquitination of metastasis suppressor 1 (MTSS1), an important tumor suppressor, mediated by the skp1-cullin1-F-box beta-transducin repeat-containing protein (SCF β-TRCP) E3 ubiquitin ligase complex, is essential for regulating cell proliferation and migration in breast and prostate cancers [12]. Additionally, there is a significantly higher phosphorylation level of basal endothelial nitric oxide synthase (eNOS) at S1177 in type 2 diabetes mellitus (T2DM) patients, and the abnormal eNOS activation mediated by phosphorylation has been suggested to play a potential role in endothelial insulin resistance [13]. Moreover, the T313M mutation of PINK1, an important regulator of mitochondrial trafficking, abolishes its phosphorylation by a protein kinase MARK2, exhibits toxic effects in neurons, and is involved in neurodegeneration in Parkinson’s disease (PD) [14]. These studies indicate that there are strong associations between PTMs and diseases. Therefore, an integrative resource of PTM–disease associations (PDAs) would be a great help for both academic research and biomedical applications.

A number of public databases have been developed for collecting and maintaining PDAs, such as KinMutBase [15], MoKCa [16], KIDFamMap [17], and HHMD [18]. The KinMutBase is the first comprehensive database, which contains 582 disease-causing mutations in 20 tyrosine and 13 serine/threonine kinase domains, with the corresponding disease types and numbers of affected patients and families available for each entry [15]. However, KinMutBase only contained mutations in protein kinase domains. To further consider mutations in full-length sequences of protein kinases, Richardson et al*.* developed the database MoKCa, which contains thousands of somatic mutations in protein kinases identified in cancers [16]. The structural and functional annotations of potential phenotypic alterations are also provided for these cancer mutations. To demonstrate the inhibitor selectivity of protein kinases in disease therapy, the integral database KIDFamMap was developed about the kinase–inhibitor interactions between 399 human protein kinases and 35,788 kinase inhibitors in 339 diseases, providing information for the inhibitor selectivity and binding mechanisms of kinases [17]. These three databases mentioned above are mainly focused on phosphorylation and protein kinases. Beyond phosphorylation, HHMD serves as an integral resource for the annotation of histone modifications in cancers, which includes 43 location-specific histone modifications in 9 human cancer types [18]. Since these databases are constructed primarily on specific PTM types or specific proteins, a general data resource for PTM–disease associations will be more advantageous for further investigations on PTMs and related diseases.

In this work, we first collected 1950 known PDAs in 749 proteins for 23 PTM types and 275 disease types from the literature. For convenience, we classified the 23 PTM types and 275 disease types into 9 and 26 super-types, respectively. Database analyses indicate that phosphorylation has the largest number of disease associations (81.49%), whereas the super-type of neurologic diseases has the largest number of associations with PTMs (18.32%). Additionally, all PDAs were classified into six types based on the PTM status in diseases, and observed that the upregulation (40.56%) and emergence (38.26%) of PTM events make up the majority of disease-associated PTM events. Moreover, by reconstructing a disease–gene network containing 749 PTM substrates, 275 diseases, and 1437 disease–gene interactions, breast cancers are found to have the largest number of associated PTMs, while the important protein kinase AKT1 has the largest numbers of connected diseases. To provide more disease information, we also integrated annotations from other public databases. Finally, we developed an integral database, PTMD, for PTMs that are associated with diseases. PTMD can serve as a useful resource for further analyzing the relations between PTMs and diseases.

## Construction and content

Here, we defined a PDA as a PTM event that is causally influenced or changed in a disease. To ensure the data quality, we first searched PubMed using multiple keywords, such as “acetylation”, “glycosylation”, “methylation”, “nitration”, “*S*-palmitoylation”, “phosphorylation”, “*S*-nitrosylation”, “sumoylation”, and “ubiquitination”. We manually curated experimentally identified PDAs from the related literature. In total, we collected 1950 PDAs in 749 proteins for 23 types of PTMs and 275 types of diseases. We also used the names of other PTM types such as malonylation and propionylation to search PubMed, but did not identify any additional PDAs.

To gain further insight into the relationships between PTMs and diseases, we classified all collected PDAs into six classes according to the status of PTM events in diseases. (i) Upregulation (U): the PTM level is upregulated in diseases. For example, the phosphorylation level of eNOS at S1177 is significantly increased in T2DM [13]. (ii) Down-regulation (D): the PTM level is down-regulated in diseases. For instance, a significant decrease in the phosphorylation level at tyrosine 216 of GSK3β, an important serine/threonine kinase, was detected in squamous cell carcinoma (SCC) samples compared to normal tissues, suggesting a pathological role of the inactivated form of GSK3β in skin carcinogenesis [19]. (iii) Presence (P): the presence of a PTM event is associated with disease progression. For example, the SCF β-TRCP-mediated ubiquitination of MTSS1 plays a regulatory role in the proliferation and migration of cancer cells [8]. (iv) Absence (A): the absence of a PTM event is involved in disease progression. For example, a S392 nonphosphorylated form of p53 is present in human breast tumors, while the inability to phosphorylate p53 at this site was related to treatment response [20], [21]. (v) Creation (C): a mutation event (single amino acid or indel mutations) creates one or multiple PTM sites or increases the protein PTM level in diseases. As an example, Fang et al*.* reported a mutant form of FAK with deletion of exon 33, which strongly increases its phosphorylation level at Y397, alters the downstream signaling, and further enhances cell migration and invasion in breast cancers [22]. (vi) Disruption (N): a mutation event that disrupts one or multiple PTM sites or reduces the protein PTM levels in diseases. A typical instance of this type of disease-associated PTMs is that the T313M mutation of PINK1 disrupts its MARK2-mediated phosphorylation and is implicated in PD [14]. Thus, the former two types (U and D) focused on the changes of PTM levels in diseases compared to normal tissues, whereas the type P and A showed whether the presence or absence of a PTM event is related to disease progression. The last two types C and N underscored the impact of a mutation event on PTM in diseases.

**Figure 1:**
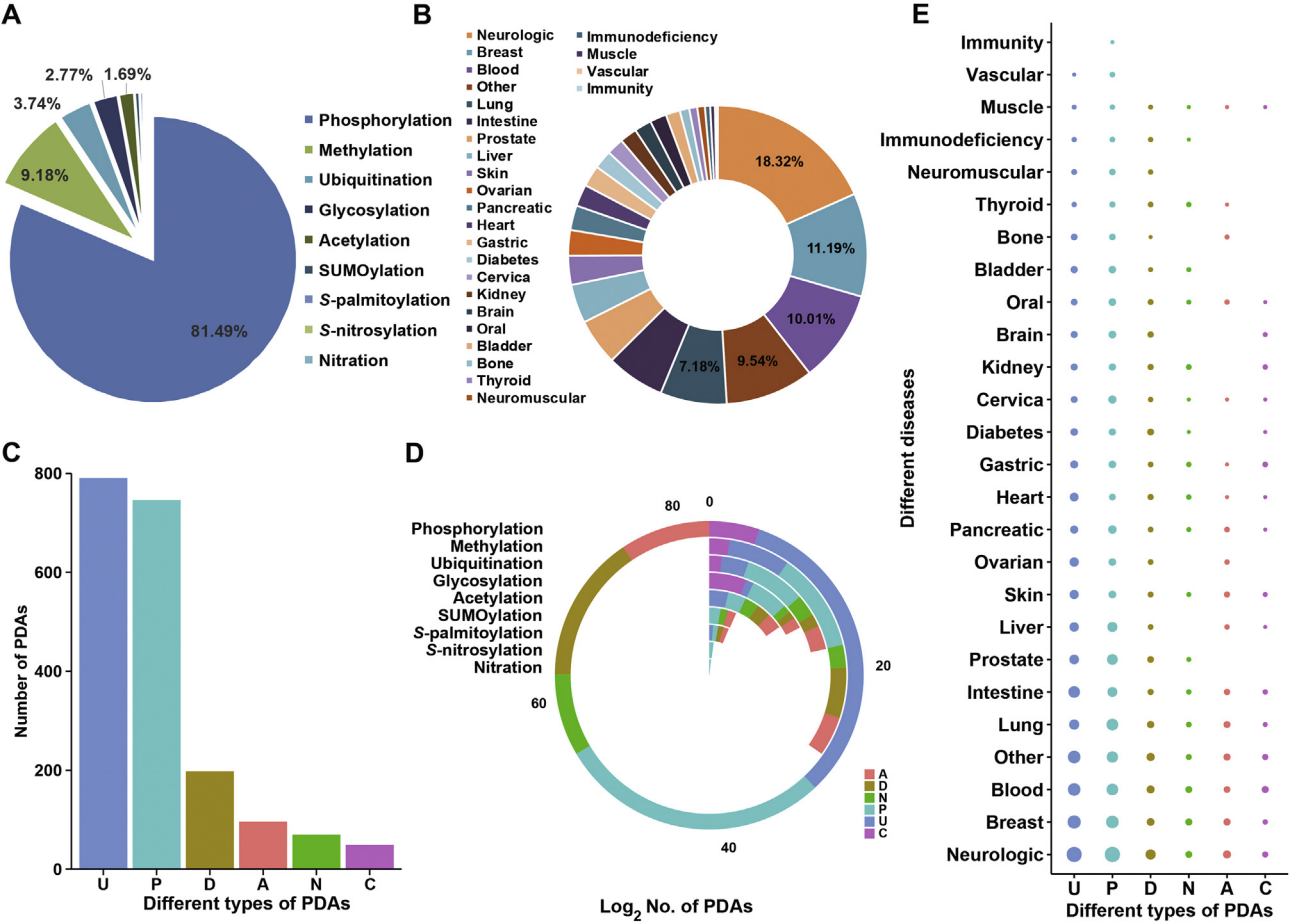
**The data in the PTMD database** **A.** The percentage of different PTM super types that are associated with diseases. In PTMD, 23 types of PTMs were classified into 9 super-types. **B.** The percentage of different super-types of diseases in PTMD. In total, 275 types of diseases are classified into 26 super-types based on the tissue information. **C.** The number of disease associations within each type of disease-associated PTMs. **D.** The number of disease associations within each type of PDAs for each PTM super-type. Blocks with different colors represent different types of PDAs, while the block length represents the number of PDAs for each PTMs. **E.** The distribution of the six types of PDAs for each disease super-type. The dot size represents the number of PDAs for each disease. U and D indicate that the PTM level is upregulated and downregulated in diseases, respectively; P and A indicate that the presence and absence of a PTM event is associated with disease progression, respectively. C indicates that a mutation event (single amino acid or indel mutations) creates one or multiple PTM sites or increases the protein PTM level in diseases, whereas N indicates that a mutation event disrupts one or multiple PTM sites or reduces the protein PTM levels in diseases. PTM, posttranslational modification; PDA, PTM–disease association.

Next, we grouped 23 PTM types into 9 super-types, including acetylation, glycosylation, methylation, nitration, *S*-palmitoylation, phosphorylation, *S*-nitrosylation, sumoylation, and ubiquitination (Figure 1A). For example, both serine/threonine phosphorylation and tyrosine phosphorylation were classified into the super-type of phosphorylation, while the monoubiquitination and polyubiquitination were classified into the super-type of ubiquitination. In our results, the top three super-types of PTMs with largest number of disease associations are phosphorylation (81.49%), methylation (9.18%), and ubiquitination (3.74%), whereas other PTM super-types only have a small number of disease associations (Figure 1A). In this regard, more efforts are required to identify these disease-associated PTMs. We also grouped 275 types of diseases into 26 super-types based on the tissue location (Figure 1B). Among all the diseases, the super-type of neurologic diseases has the largest number of associations with PTMs (18.32%) (Figure 1B). This observation is consistent with the mainstream viewpoint that abnormal PTM events extensively occur in numerous neurodegenerative diseases, such as Alzheimer's disease (AD) and PD, and are involved in neuronal dysfunction and cell death [11], [23], [24]. The second and third super-types of disease with the largest number of PTM associations are breast (11.19%) and blood (10.01%) diseases (Figure 1B). Among the six types of PDAs, we found that 40.56% and 10.15% PTMs are upregulated and down-regulated in diseases, respectively (Figure 1C), suggesting that an increase in PTM levels may contribute more to disease development. Their potentials as candidate biomarkers for disease diagnosis and drug design warrant further studies. Moreover, 38.26% of disease-associated PTM events fall into the type P, while only 4.92% disease-associated PTMs are classified in the type A (Figure 1C), indicating that PTM events preferentially exists during disease progression, whereas the development of only a small proportion of diseases is associated with the loss of PTMs. It is of note that within each type of PDAs, the numbers of disease associations varied for different super-types of PTMs (Figure 1D). For instance, more than 700 disease-associations were found for the phosphorylation in the type U, and 559 relations are in the type P (Figure 1D). Additionally, the numbers of disease associations within each type of PDAs varied remarkably among 26 super-types of diseases (Figure 1E).

After collecting and classifying the PDAs, we further searched the UniProt database [25] to obtain the corresponding protein sequences and the annotation information. To provide more disease information for each protein, we integrated the knowledge from several public databases, including Cancer Gene Census [26], Comparative Toxicogenomics Database (CTD) [27], DisGeNET [28], and OMIM [29]. Additionally, we integrated experimentally identified PTM sites from our previous studies [30], [31]. The protein–protein interaction (PPI) information was also obtained from public databases, including BioGRID [32], IntAct [33], MINT [34], HPRD [35], and DIP [36]. The PPI networks of disease-associated genes/proteins were visualized using Cytoscape Web v1.0.4 interface [37]. The online service of PTMD database was implemented in PHP + MySQL + JavaScript and is freely accessible at http://ptmd.biocuckoo.org.

## Usage

**Figure 2:**
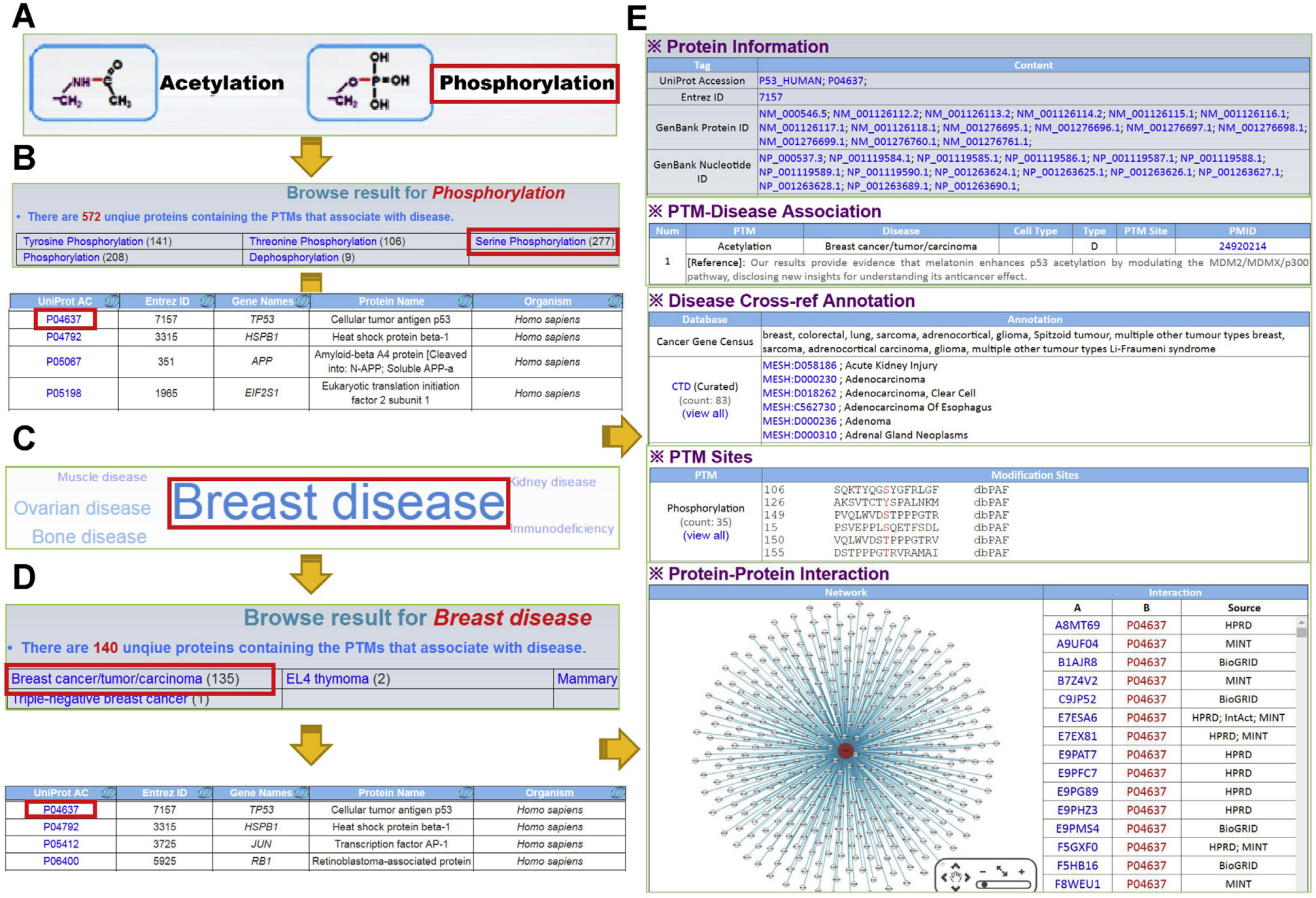
**The browse options of PTMD** We provided two options to browse the database: by PTMs (**A**) and by diseases (**C**). **B.** All phosphorylated substrates with different PTM types of phosphorylation (tyrosine, threonine, and serine phosphorylation) are shown in a tabular format. **D.** All specific types of breast diseases, such as breast cancer and mammary tumor, are shown. **E.** The detailed information of human p53 in PTMD database, including Protein Information, PTM–Disease Association, Disease Cross-ref Annotation, PTM Sites, and Protein–Protein Interaction.

The PTMD database was developed in an easy-to-use mode. Here, we took human p53 as an example to demonstrate the use of PTMD. The Browse page is the major site for users to look through the PTMD, where data can be browsed in two different ways, *i.e.*, by PTM types and by diseases (Figure 2). For convenience, the 9 super types of PTMs were present on the left side (Figure 2A), whereas the 26 super-types of diseases were visualized in a tag cloud picture on the right side of the Browse page (Figure 2C). For the option “Browse by PTMs”, after clicking the icon phosphorylation (Figure 2A), the corresponding PTM types, including dephosphorylation, serine phosphorylation, threonine phosphorylation, and tyrosine phosphorylation, together with all protein substrates, can be displayed (Figure 2B). For the option “Browse by Disease”, users can select a disease super-type by clicking the tissue name in the tag cloud (Figure 2C). Specifically, the larger font size indicates the larger number of PDAs. By clicking the link “Breast disease”, all specific types of breast diseases can be shown in a tabular format (Figure 2D). Additionally, users can click the “Breast cancer/tumor/carcinoma” link to visualize all PTM substrates that are associated with breast cancers (Figure 2D). Then, by clicking the UniProt accession number of “P04637”, all PDAs for p53 can be shown (Figure 2E).

**Figure 3:**
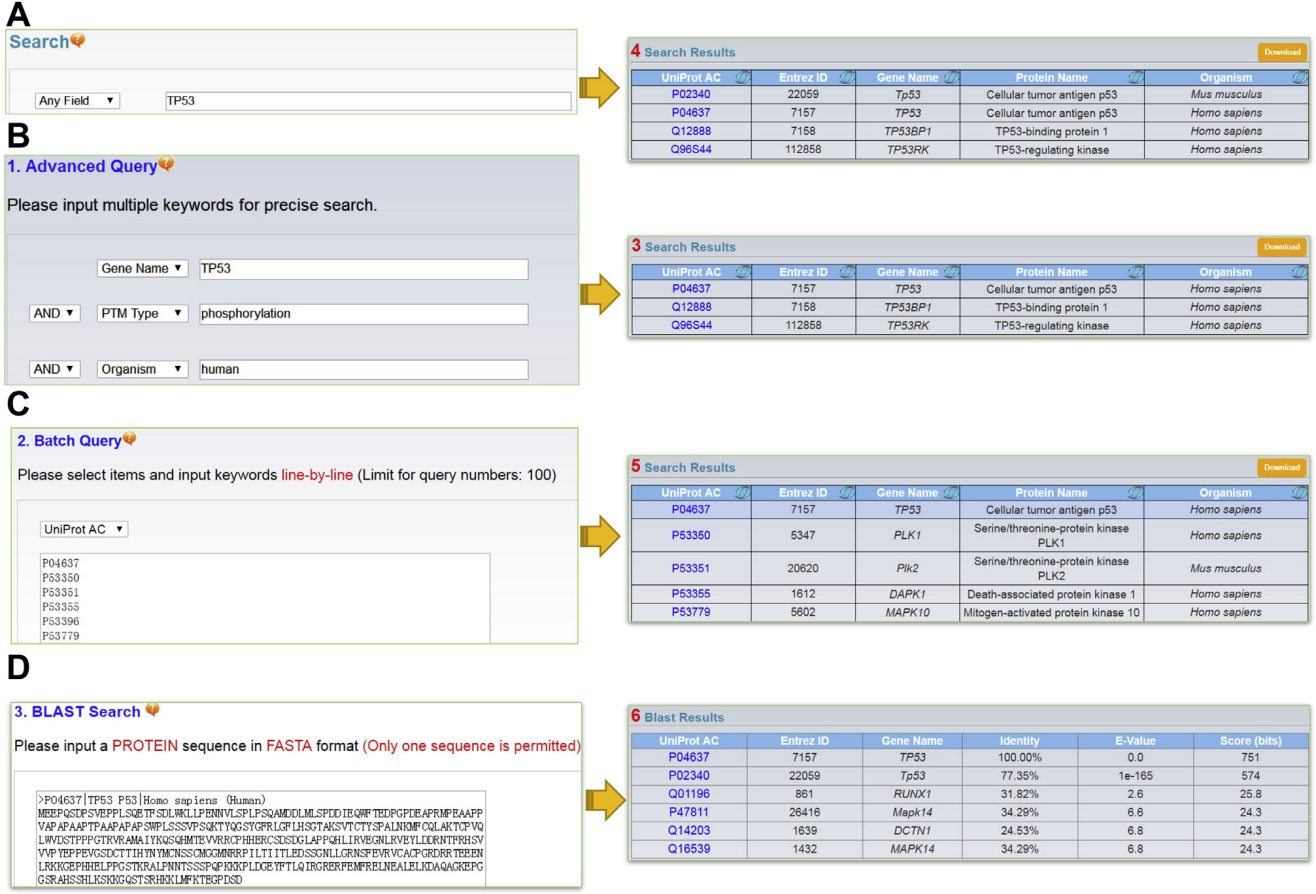
**The search and advance options** **A.** The database can be directly searched with one or multiple keywords. **B.** Advanced query allows users to input up to three search terms. **C.** Batch query allows users to query multiple keywords in a line-by-line format. **D.** Blast search option interrogates a protein sequence for detecting identical or homologous sequences.

On the homepage, users can directly search the PTMD database by inputting one or multiple keywords (Figure 3A). For example, if the keyword “p53” is submitted, the corresponding results for p53 is shown in a tabular format, including UniProt accessions, Entrez gene IDs, protein names, and gene names (Figure 3A). Moreover, the database provides three additional advanced options, namely, (i) Advanced query, (ii) Batch query, and (iii) BLAST search. (i) Advanced query. In this option, users could use relatively complex and combined keywords to locate the precise information, with up to three search terms. The interface of search engine allows the querying by different database fields and the linking of queries through three operators “and”, “or”, and “not” (Figure 3B). (ii) Batch query. Users can enter multiple keywords, such as UniProt accession numbers, gene IDs, gene names, PTM types, diseases, and tissues, in a line-by-line format for querying (Figure 3C). Again, all related PTM substrates can be presented in a tabular page. (iii) BLAST search. This option was designed for querying the related information in PTMD by protein sequences. The blastall program of NCBI BLAST packages [38] was included in the PTMD database. Users can enter a protein sequence in FASTA format for searching identical or homologous proteins (Figure 3D).

## Discussion

After the first phosphorylated protein casein was discovered in 1883, the identification and characterization of protein PTMs has been pursued for over 135 years [39]. Extensive experimental studies have revealed that the abnormal PTM status is frequently involved in disease development and progression [9], [10], [11]. Although a number of public databases have been constructed for PDAs [15], [16], [17], [18], these databases are mainly focused on specific PTM types or specific proteins. Thus, it is still not clear how many aberrant PTM events are associated with human diseases, and a comprehensive and integrated data resource of disease-associated PTMs with detailed annotations would offer great help for both academic research and biomedical applications.

**Figure 4:**
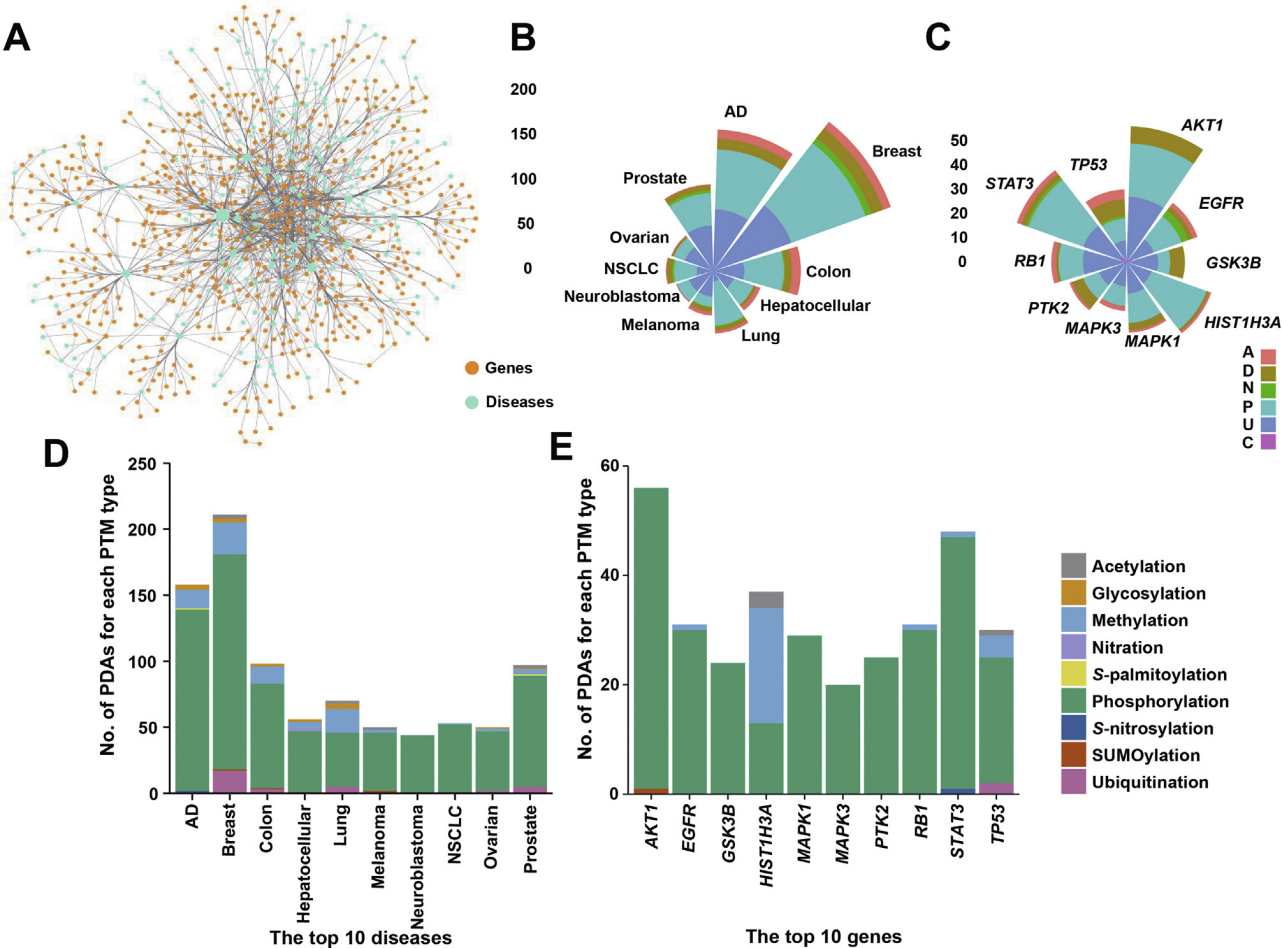
**A disease–gene network** **A.** The network was visualized with Cytoscape v3.6.0 [47]. **B.** The top ten diseases with the largest number of associations with PTM substrates. **C.** The top ten genes with the largest number of associated diseases. **D.** The number of each PTM super-type in the top ten diseases. **E.** The numbers of each PTM super-type for the top ten genes. AD, Alzheimer’s disease; NSCLC, non-small-cell lung cancer.

In total, our PTMD database contains 1950 PDAs in 749 proteins for 23 PTM types and 275 diseases. For a better understanding of the relationships between PTM substrates and diseases, we also constructed a disease–gene network. As previously described [40], a gene was connected with a disease if a PDA links them. The network contains 749 PTM proteins and 275 diseases together with 1437 disease–gene interactions (Figure 4A). Obviously, we find that a large proportion of diseases are associated with only a small number of genes, whereas a considerable number of diseases are associated with many genes (Figure 4A). From the network, the top ten diseases with the largest number of relations to genes and the top ten genes with the largest number of connected diseases were shown (Figure 4B, C), with the numbers of associations for each PTM super-type also counted (Figure 4D, E). In the results, the distribution of the six types of PDAs varied in these diseases and genes, and the upregulation and presence of PTM events dominate these influences. Among diseases, the breast cancers have the highest number of associated PTMs (Figure 4D), and at least three different super-types of PTMs are implicated in most of these diseases. Especially, for breast cancers, prostate cancers, and Alzheimer's disease, up to six different associated PTM super-types were found. The results indicate that multiple PTMs may crosstalk with each other and interact in the development of complex diseases. For example, the K167 ubiquitination and T166 phosphorylation of c-FLIP, an apoptosis inhibitor, were both found in prostate cancer [41]. T166 phosphorylation is required for the K167 ubiquitination, which further regulates the stability of c-FLIP and influences the sensitivity of cancer cells to apoptosis [41]. Additionally, the K810 methylation of the tumor suppressor protein RB1 increases the phosphorylation level of adjacent S807/S811, which further enhances the cell cycle progression of bladder cancers [42]. The top ten genes with the largest number of connected diseases were displayed, and almost all of these genes are involved in key signaling pathways (Figure 4C). For example, the serine/threonine protein kinase AKT1, which has the largest number of associated diseases [35] in the network, is implicated in PI3K/AKT signaling, thus contributing to the cell cycle progression and cellular growth [43]. Additionally, STAT3, a signal transducer and activator of transcription that plays an important role in JAK2/STAT3 signaling, is connected with 33 types of diseases and is essential for the regulation of cell growth and proliferation [44]. In addition, we found p53 is connected with 21 types of cancers and neurologic diseases, and the phosphorylation at S46 induces apoptotic cell death by enhancing the protein stability and transcriptional activity of p53 [45], [46]. The down-regulation of p53 phosphorylation at S46 maintains tumorigenesis in cancers [45] but is upregulated by mutant huntingtin (mHtt) to promote neuronal apoptosis in Huntington’s disease (HD) [46]. Thus, one PTM event can have different states and be classified into different classes based on associated diseases.

Taken together, our analyses confirm a strong relation between PTMs and diseases, and the results are consistent with the experimental observations. Additionally, the PTMD database can serve as a useful resource for further analyzing the relationships between PTMs and diseases. This data resource will be updated twice per year, as new disease-associated PTMs become available.

## Authors' contributions

YX and QC conceived, designed, and supervised the study. HX and YW performed the analysis and developed the database. SL, WD, and DP contributed to data analysis. YX and HX wrote the manuscript. All authors read and approved the final manuscript.

## Competing interests

The authors have declared no competing interests.
